# JAVEMACS: a real-world study of avelumab maintenance therapy for advanced urothelial carcinoma in Japan

**DOI:** 10.1016/j.esmorw.2025.100646

**Published:** 2025-11-20

**Authors:** H. Kitamura, T. Kobayashi, Y. Endo, M. Ikeda, K. Yonemori, M. Nakayama, A. Fujihara, T. Abe, F. Shimizu, K. Fujimoto, T. Nakagawa, S. Hatakeyama, K. Murakami, K. Nishihara, D. Ikarashi, N. Masumori, S. Naito, K. Fujita, N. Hayakawa, T. Hara, N. Miura, T. Negishi, J. Yatsuda, M. Kobayashi, N. Nishiyama, R. Taoka, J. Furukawa, M. Shono, S. Shirotake, E. Kikuchi

**Affiliations:** 1 Department of Urology, Faculty of Medicine, University of Toyama, Toyama, Japan; 2 Department of Urology, Kyoto University Graduate School of Medicine, Kyoto, Japan; 3 Department of Urology, Nippon Medical School Hospital, Tokyo, Japan; 4 Department of Urology, Kitasato University School of Medicine, Kanagawa, Japan; 5 Department of Medical Oncology, National Cancer Center Hospital, Tokyo, Japan; 6 Department of Urology, Osaka International Cancer Institute, Osaka, Japan; 7 Department of Urology, Kyoto Prefectural University of Medicine, Kyoto, Japan; 8 Department of Renal and Genitourinary Surgery, Hokkaido University Graduate School of Medicine, Hokkaido, Japan; 9 Department of Urology, Juntendo University Graduate School of Medicine, Tokyo, Japan; 10 Department of Urology, Nara Medical University, Nara, Japan; 11 Department of Urology, Teikyo University School of Medicine, Tokyo, Japan; 12 Department of Urology, Hirosaki University Graduate School of Medicine, Aomori, Japan; 13 Department of Urology, Kurume University School of Medicine, Fukuoka, Japan; 14 Department of Urology, Iwate Medical University School of Medicine, Iwate, Japan; 15 Department of Urology, Sapporo Medical University School of Medicine, Hokkaido, Japan; 16 Department of Urology, Yamagata University, Faculty of Medicine, Yamagata, Japan; 17 Department of Urology, Kindai University, Faculty of Medicine, Osaka, Japan; 18 Department of Urology, St. Marianna University School of Medicine, Kanagawa, Japan; 19 Division of Urology, Kobe University Graduate School of Medicine, Kobe, Japan; 20 Department of Urology, Ehime University Graduate School of Medicine, Ehime, Japan; 21 Department of Urology, National Hospital Organization Kyushu Cancer Center, Fukuoka, Japan; 22 Department of Urology, Graduate School of Medical Sciences, Kumamoto University, Kumamoto, Japan; 23 Department of Urology, Akita University Graduate School of Medicine, Akita, Japan; 24 Department of Urology, Faculty of Medicine, Kagawa University, Kagawa, Japan; 25 Department of Urology, Tokushima University Graduate School of Biomedical Sciences, Tokushima, Japan; 26 Medical Department, Merck Biopharma Co., Ltd., Tokyo, Japan, an affiliate of Merck KGaA; 27 Department of Uro-Oncology, Saitama Medical University International Medical Center, Saitama, Japan

**Keywords:** avelumab, bladder cancer, maintenance, real-world study, urothelial carcinoma

## Abstract

**Background:**

Avelumab maintenance therapy was approved in Japan in February 2021 for patients with unresectable locally advanced or metastatic urothelial carcinoma (la/mUC) without disease progression after platinum-based chemotherapy (PBC). We report the primary analysis from the JAVEMACS chart review study of avelumab maintenance therapy in Japan.

**Materials and methods:**

This multicenter, retrospective study collected data from medical charts of patients with la/mUC who received avelumab maintenance after first-line (1L) PBC.

**Results:**

Between February 2021 and December 2023, 350 patients received avelumab maintenance; median age was 73 years and most were male (74.0%). Prior 1L PBC was cisplatin–gemcitabine in 56.0% and carboplatin–gemcitabine in 33.1%; 28.6% were cisplatin eligible and 52.9% were cisplatin ineligible/platinum eligible. At data cut-off (June 2024), 67 patients (19.1%) were still receiving avelumab. Among 283 patients who discontinued avelumab, 200 (70.7%) received second-line (2L) treatment. In the overall population, median overall survival (OS) from avelumab initiation was 31.8 months [95% confidence interval (CI) 24.6 months-not estimable (NE)]. In subgroups who received 2L enfortumab vedotin (EV) (*n* = 133), PBC (*n* = 41), or pembrolizumab (*n* = 17), median OS from the start of 2L treatment was 17.8 months (95% CI 11.9 months-NE), 15.1 months (95% CI 11.6 months-NE), and 15.6 months (95% CI 8.6-21.3 months), respectively. Limitations include the study’s retrospective nature.

**Conclusions:**

Results from JAVEMACS confirm the effectiveness of avelumab maintenance in real-world clinical practice in Japan, supporting its recommendation for patients with la/mUC without disease progression following 1L PBC. This treatment sequence followed by 2L EV appeared to be associated with encouraging long-term outcomes in this real-world population.

## Highlights

• Avelumab 1L maintenance is recommended for eligible patients with advanced UC.
• JAVEMACS is a real-world chart review of avelumab 1L maintenance in Japan (*N* = 350).
• In JAVEMACS, median OS with avelumab maintenance was 31.8 months.
• This treatment sequence followed by 2L EV had promising long-term outcomes.
• These data support the use of avelumab 1L maintenance in eligible patients in Japan.

## Introduction

Bladder cancer is the ninth most common cancer globally, with 614 298 new cases and 220 596 deaths in 2022.^1^ For patients with metastatic disease, the 5-year survival rate was <10%.^2^ Urothelial carcinoma (UC) accounts for >90% of bladder cancer cases.^3^ UC can also occur in the upper urinary tract, including the ureter and renal pelvis.^4^

Avelumab, an anti-programmed death-ligand 1 immune checkpoint inhibitor, was approved in Japan on 24 February 2021 as maintenance therapy for patients with unresectable locally advanced/metastatic (la/m) UC without disease progression following platinum-based chemotherapy (PBC) and is recommended in clinical practice guidelines published by the Japanese Urological Association.^5^^,^^6^ Approval was based on the JAVELIN Bladder 100 phase III trial in which avelumab first-line (1L) maintenance + best supportive care (BSC) significantly prolonged overall survival (OS) and progression-free survival (PFS) versus BSC alone in patients with unresectable la/mUC that had not progressed with 1L PBC.^7^ After ≥2 years of follow-up in all patients, median OS was 23.8 versus 15.0 months [hazard ratio (HR) 0.76, 95% confidence interval (CI) 0.63-0.91, *P* = 0.0036], and median PFS was 5.5 versus 2.1 months (HR 0.54, 95% CI 0.46-0.64, *P* < 0.0001), respectively.^8^ Avelumab 1L maintenance also demonstrated acceptable long-term safety. In a *post hoc* analysis of patients enrolled in Japan (*n* = 73), efficacy and safety findings were generally consistent with the overall trial population; in the avelumab + BSC versus BSC-alone arms, median OS was 24.7 versus 18.7 months (HR 0.81, 95% CI 0.41-1.58), and median PFS was 5.6 versus 1.9 months (HR 0.63, 95% CI 0.36-1.11), respectively.^9^ Following approval of avelumab maintenance in Japan, a prospective post-marketing surveillance study was conducted, which demonstrated the real-world effectiveness and safety of avelumab in patients with unresectable la/mUC in Japan (*N* = 453).^10^ For patients with disease progression during 1L treatment, pembrolizumab and enfortumab vedotin (EV) have been approved in Japan as subsequent treatment options.

We report results from the JAVEMACS chart review study of patients with la/mUC who were treated with avelumab maintenance in Japan.

## Materials and methods

### Study design and patient population

JAVEMACS (NCT06412848) was a multicenter, noninterventional, retrospective medical chart review of patients in Japan. The study included all patients at participating centers who met the eligibility criteria. Eligible patients were aged ≥18 years, had la/mUC that had not progressed [ongoing stable disease (SD), partial response (PR), or complete response (CR)] following 1L PBC, and initiated their first dose of avelumab maintenance therapy between 24 February 2021 and December 2023. To ensure a minimum observation period, patients were required to have received their first avelumab dose at least 6 months before the date of study approval at each site. Patients who participated in a clinical trial in la/mUC during the study period were excluded. Data were collected retrospectively, and the follow-up period was defined as the period from the start of avelumab maintenance therapy until loss to follow-up, death due to any cause, or the end of the data collection period, whichever occurred first. Characteristics of 1L PBC were also collected. Eligibility for cisplatin or platinum was determined per published criteria, using data collected at start of 1L PBC. Cisplatin ineligibility was defined as a patient who met any of the following criteria: Eastern Cooperative Oncology Group performance status (ECOG PS) ≥2, creatinine clearance (CrCl) of <60 ml/min, grade ≥2 hearing loss, grade ≥2 peripheral neuropathy, and/or class III or worse New York Heart Association heart failure.^11^ Platinum ineligibility was defined as a patient who met any of the following criteria: ECOG PS ≥3, CrCl of <30 ml/min, or ECOG PS of 2 and CrCl of <45 ml/min.^12^

Data were extracted by trained investigators or clinical research coordinators at each site using a standardized data dictionary (Electronic Data Capture manual); centralized data management included automated range/logic checks, query resolution, and predefined reconciliation of key variables (e.g. dates, baseline clinical characteristics, treatment lines). A subset of the data underwent dual review to assess consistency.

This study was conducted in accordance with relevant laws, the Declaration of Helsinki, and the Ethical Guidelines for Life Science and Medical Research Involving Human Subjects dated 23 March 2021 (partially revised 27 March 2023), issued by the Ministry of Education, Culture, Sports, Science and Technology, Ministry of Health, Labor and Welfare, and Ministry of Economy, Trade and Industry in Japan. All patients provided informed consent before inclusion in the study. The study protocol, amendments, and other relevant documents were approved by the independent ethics committee/institutional review board of the nonprofit organization MINS (Tokyo, Japan; Approval No. MINS-REC-240211) and hospital director of each site before starting the study.

### Objectives

The primary objectives were to describe the baseline characteristics of patients who received avelumab maintenance therapy, characteristics of 1L PBC before avelumab maintenance, and subsequent treatments following avelumab maintenance. The secondary objective was to evaluate outcomes of avelumab maintenance and subsequent therapies, including OS (defined as time from start of therapy to death due to any cause), PFS (defined as time from start of therapy to progression or death), PFS2 (defined as time from start of therapy to progression with next-line treatment or death), objective response rate (ORR), and disease control rate (DCR; rate of CR, PR, SD, and non-CR/non-progressive disease as best response) assessed by the physician per the Response Evaluation Criteria in Solid Tumors version 1.1. Exploratory analyses included survival analyses in subgroups of interest and a multivariate analysis to evaluate predictive factors for OS.

### Statistical analysis

This study was descriptive in nature; therefore, sample size calculations were not applicable. Two analysis sets were included: the full analysis set, including all patients who received avelumab maintenance therapy, and the second-line (2L) therapy analysis set, including all patients who also received 2L treatment. Continuous variables were summarized using descriptive statistics, and categorical variables were summarized by frequency counts and percentages. Time-to-event analyses were conducted using the Kaplan–Meier method, and corresponding 95% CIs were calculated using the Greenwood formula. A multivariate Cox regression analysis of OS across subgroups was carried out to calculate HRs and corresponding 95% CIs, and *P* values were calculated using the Wald test. All analyses were carried out using SAS® software version 9.4 (SAS Institute Inc, Cary, NC) or later.

## Results

### Study population

**Figure 1:**
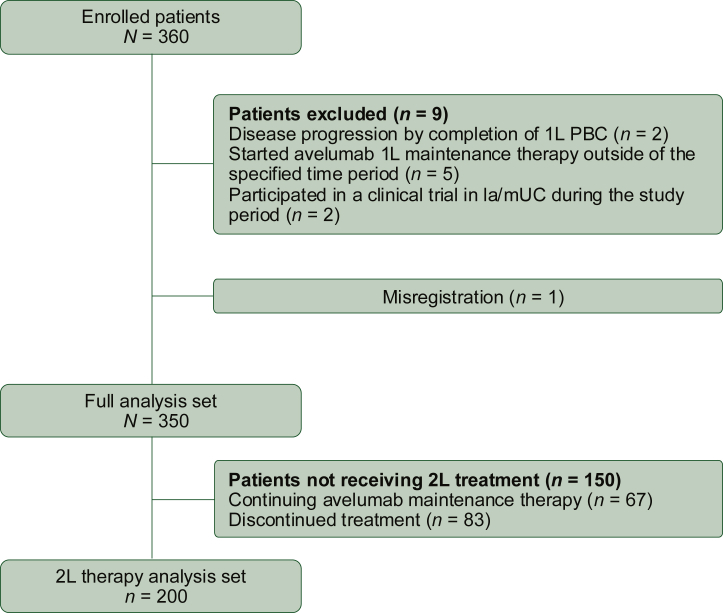
**Patient disposition.** 1L, first line; 2L, second line; la/mUC, locally advanced/metastatic urothelial carcinoma; PBC, platinum-based chemotherapy.

Between February 2021 and December 2023, 350 patients at 26 centers, including university hospitals and cancer institutes, had received a first dose of avelumab maintenance therapy and met the study’s eligibility criteria (Figure 1). At the start of avelumab maintenance, median age was 73 years [interquartile range (IQR) 67-78 years]; 57 patients (16.3%) were aged ≥80 years (Table 1). Most patients were male [*n* = 259 (74.0%)] and had an ECOG PS of 0 [*n* = 284 (81.1%)]. The primary tumor location was the bladder in 177 patients (50.6%) and the ureter or renal pelvis in 168 (48.0%). Characteristics in patients who received 2L treatment were similar to the overall population (Supplementary Table S1, available at https://doi.org/10.1016/j.esmorw.2025.100646), although a lower proportion of patients were aged ≥80 years (11.5% versus 16.3%). Differences in laboratory findings at the start of avelumab maintenance treatment versus the start of 1L PBC (Supplementary Table S2, available at https://doi.org/10.1016/j.esmorw.2025.100646) included a higher proportion of patients with hemoglobin levels of <10 g/dl (34.6% versus 12.9%), and a lower proportion with a neutrophil-to-lymphocyte ratio ≥3 (34.9% versus 46.9%) and C-reactive protein (CRP) levels of >0.3 mg/dl (31.7% versus 50.9%). There were no notable changes in CrCl, ECOG PS, or body mass index (BMI) between start of 1L PBC and start of avelumab maintenance.

**Table 1:** Baseline characteristics at the start of avelumab maintenance therapy

	Overall population (*N* = 350)
Age, years	
Median age (IQR)	73 (67-78)
<65, *n* (%)	61 (17.4)
≥65 and <75, *n* (%)	145 (41.4)
≥75 and <80, *n* (%)	87 (24.9)
≥80, *n* (%)	57 (16.3)
Sex, *n* (%)	
Male	259 (74.0)
Female	91 (26.0)
BMI, kg/m^2^	
Median BMI (IQR)	23.0 (20.9-24.9)
<18.5, *n* (%)	20 (5.7)
≥18.5 and <25, *n* (%)	229 (65.4)
≥25, *n* (%)	76 (21.7)
Unknown, *n* (%)	25 (7.1)
Smoking status, *n* (%)	
Ever smoker (current or former) includes all tobacco	228 (65.1)
Never smoker	110 (31.4)
Unknown	12 (3.4)
ECOG PS, *n* (%)	
0	284 (81.1)
1	56 (16.0)
≥2	6 (1.7)
Unknown	4 (1.1)
Primary tumor location, *n* (%)	
Ureter or renal pelvis	168 (48.0)
Bladder	177 (50.6)
Urethra	5 (1.4)
Metastatic site, *n* (%)	
Any	293 (83.7)
Regional lymph node	184 (52.6)
Distant lymph node	105 (30.0)
Visceral	113 (32.3)
Lung	75 (21.4)
Liver	24 (6.9)
Peritoneum	14 (4.0)
Other organs	10 (2.9)
Bone	55 (15.7)
Other	12 (3.4)
Hemoglobin, g/dl	
Median (IQR)	10.5 (9.6-11.5)
<10, *n* (%)	121 (34.6)
≥10, *n* (%)	224 (64.0)
Unknown, *n* (%)	5 (1.4)
NLR	
Median (IQR)	2.47 (1.70-3.54)
<3, *n* (%)	221 (63.1)
≥3, *n* (%)	122 (34.9)
Unknown, *n* (%)	7 (2.0)
CRP, mg/dl	
Median (IQR)	0.16 (0.07-0.46)
≤0.3, *n* (%)	227 (64.9)
>0.3, *n* (%)	111 (31.7)
Unknown, *n* (%)	12 (3.4)
Creatinine clearance, ml/min	
Median (IQR)	50.9 (39.9-64.0)
≥60, *n* (%)	109 (31.1)
<60, *n* (%)	224 (64.0)
Unknown, *n* (%)	17 (4.9)
Radical surgery history, *n* (%)	
Yes	140 (40.0)
No	210 (60.0)
Timing of avelumab initiation, *n* (%)	
2021	115 (32.9)
≥2022	235 (67.1)

BMI, body mass index; CRP, C-reactive protein; ECOG PS, Eastern Cooperative Oncology Group performance status; IQR, interquartile range; NLR, neutrophil-to-lymphocyte ratio.

### 1L PBC treatment

In the overall population at the start of 1L PBC, 100 patients (28.6%) were classified as cisplatin eligible, 185 (52.9%) as cisplatin ineligible/platinum eligible, and 30 (8.6%) as platinum ineligible (Table 2). Prior 1L PBC regimen was cisplatin + gemcitabine in 196 patients (56.0%), carboplatin + gemcitabine in 116 (33.1%), and dose-dense methotrexate, vinblastine, doxorubicin, and cisplatin (ddMVAC) in 32 (9.1%). Number of PBC cycles received was 1-3 in 68 patients (19.4%), 4 in 205 (58.6%), 5 or 6 in 61 (17.4%), and ≥7 in 16 (4.6%). Platinum dose was reduced in 114 patients (32.6%), with the first reduction mostly occurring in the first (14.6%) or second cycle (10.9%). Best overall response to 1L PBC was CR in 32 patients (9.1%), PR in 180 (51.4%), and SD in 138 (39.4%). Median time from start of PBC to start of avelumab was 19.3 weeks (IQR 15.4-24.1 weeks). Median time from end of PBC to start of avelumab was 5.1 weeks (IQR 3.6-7.1 weeks) and was <4 weeks in 95 patients (27.1%), 4-10 weeks in 218 (62.3%), and >10 weeks in 36 (10.3%). In patients who received 2L treatment, characteristics of 1L PBC were similar to the overall population (Supplementary Table S3, available at https://doi.org/10.1016/j.esmorw.2025.100646).

**Table 2:** Characteristics of 1L PBC before avelumab maintenance

	Overall population (*N* = 350)
Cisplatin/platinum eligibility, *n* (%)	
Cisplatin eligible	100 (28.6)
Cisplatin ineligible^a^ and platinum eligible	185 (52.9)
Platinum ineligible^b^	30 (8.6)
Unknown	35 (10.0)
1L PBC regimen, *n* (%)	
Cisplatin + gemcitabine	196 (56.0)
Carboplatin + gemcitabine	116 (33.1)
ddMVAC	32 (9.1)
Other^c^	6 (1.7)
Number of cycles	
Median (IQR)	4 (4-4)
1-3, *n* (%)	68 (19.4)
4, *n* (%)	205 (58.6)
5 or 6, *n* (%)	61 (17.4)
≥7, *n* (%)	16 (4.6)
Platinum dose reduction, *n* (%)	114 (32.6)
First cycle when platinum dose reduction occurred, median (IQR)	2 (1-2)
Duration of treatment, median (IQR), weeks^d^	19.3 (15.4-24.1)
Treatment-free interval, weeks	
Median (IQR)	5.1 (3.6-7.1)
<4, *n* (%)	95 (27.1)
4-10, *n* (%)	218 (62.3)
>10, *n* (%)	36 (10.3)
BOR, *n* (%)	
CR	32 (9.1)
PR	180 (51.4)
SD	138 (39.4)

1L, first line; BOR, best overall response; CR, complete response; CrCl, creatinine clearance; ddMVAC, dose-dense methotrexate, vinblastine, doxorubicin, and cisplatin; ECOG PS, Eastern Cooperative Oncology Group performance status; IQR, interquartile range; PBC, platinum-based chemotherapy; PR, partial response; SD, stable disease.

a Cisplatin ineligibility was defined as a patient who met any of the following criteria at start of 1L PBC: ECOG PS ≥2, CrCl of <60 ml/min, grade ≥2 hearing loss, grade ≥2 peripheral neuropathy, and/or class III or worse New York Heart Association heart failure (NYHA).^11^ In the study population, cisplatin ineligibility was due to CrCl <60 to ≥45 mL/min in 94 patients (50.8%), CrCl <45 to ≥30 mL/min in 87 (47.0%), ECOG PS ≥2 in 5 (2.7%), and grade ≥2 hearing impairment in 4 (2.2%). No patient had grade ≥2 peripheral neuropathy or NYHA class ≥III at start of 1L PBC. Some patients had >1 reason.

b Platinum ineligibility was defined as a patient who met any of the following criteria: ECOG PS ≥3, CrCl of <30 ml/min, or ECOG PS of 2 and CrCl of <45 ml/min.^12^ In the study population, platinum ineligibility was due to CrCl <30 mL/min in 28 patients (93.3%) and ECOG PS 3 in 2 patients (6.7%). No patient had ECOG PS 2 and CrCl of <45 ml/min.

c Other 1L PBC regimens included nedaplatin + gemcitabine (*n* = 4), cisplatin + etoposide (*n* = 1), and carboplatin + etoposide (*n* = 1).

d One patient with unknown start and end dates of 1L PBC was excluded from this analysis.

### Avelumab maintenance and subsequent treatment

At data cut-off (June 2024), median follow-up from start of avelumab was 14.6 months (IQR 9.7-23.9 months). In the overall population, median duration of avelumab treatment was 14.3 weeks (IQR 7.1-30.9 weeks), and median number of doses was 10 (IQR 5-20). At last follow-up, 200 patients (57.1%) were alive with ongoing follow-up.

**Figure 2:**
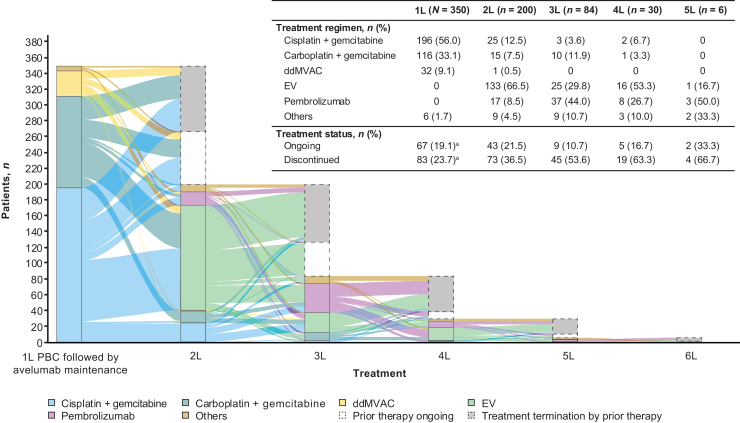
**Treatment patterns in patients treated with avelumab maintenance (*N* = 350).** 1L, first line; 2L, second line; 3L, third line; 4L, fourth line; 5L, fifth line; 6L, sixth line; ddMVAC, dose-dense methotrexate, vinblastine, doxorubicin, and cisplatin; EV, enfortumab vedotin; PBC, platinum-based chemotherapy. ^a^Treatment status refers to avelumab maintenance therapy.

At data cut-off, 67 patients (19.1%) were still receiving avelumab, 83 (23.7%) had discontinued avelumab without 2L treatment, and 200 (57.1%) had discontinued and received 2L treatment (70.7% of discontinuing patients) (Figure 2). The most common 2L treatments were EV in 133 patients (66.5%), PBC in 41 [20.5%; cisplatin + gemcitabine in 25 (12.5%), carboplatin + gemcitabine in 15 (7.5%), and ddMVAC in 1 (0.5%)], and pembrolizumab in 17 (8.5%). A higher proportion of patients who started avelumab in 2022 or later (*n* = 235) versus 2021 (*n* = 115) received 2L EV treatment (78.6% versus 43.5%). In the overall population, 84 patients (24.0%) received third-line (3L) treatment and 30 (8.6%) received fourth-line (4L), most commonly pembrolizumab (44.0% and 26.7%, respectively) and EV (29.8% and 53.3%, respectively) (Figure 2).

### Effectiveness

**Figure 3:**
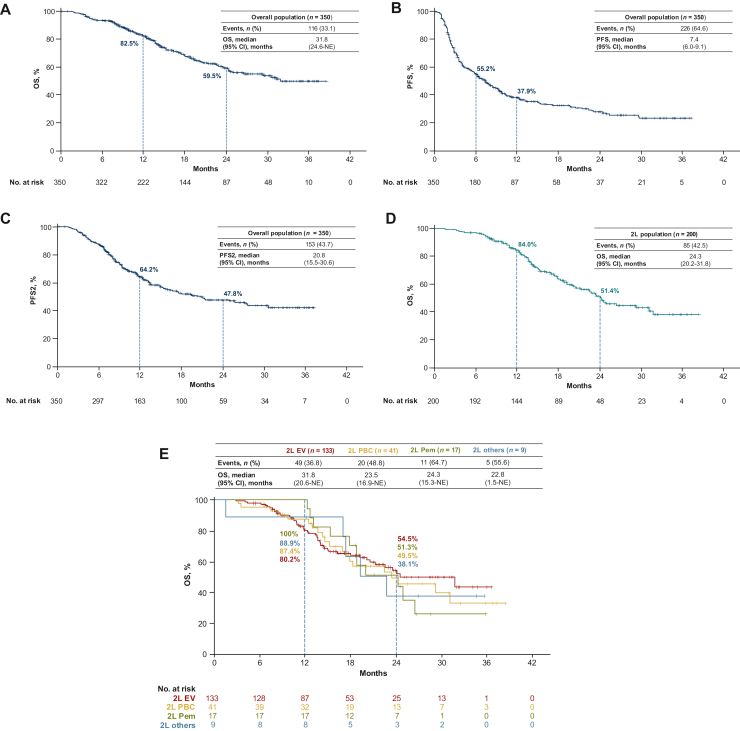
**Outcomes from the start of avelumab maintenance.** (A) OS in the overall population. (B) PFS in the overall population. (C) PFS2 in the overall population. (D) OS in patients who received 2L treatment. (E) OS in subgroups defined by 2L treatment. 2L, second line; CI, confidence interval; EV, enfortumab vedotin; NE, not estimable; OS, overall survival; PBC, platinum-based chemotherapy; Pem, pembrolizumab; PFS, progression-free survival; PFS2, progression-free survival 2 (time to progression with 2L treatment or death).

In the overall population, median OS from start of avelumab was 31.8 months [95% CI 24.6 months-not estimable (NE)] (Figure 3A) and median PFS was 7.4 months (95% CI 6.0-9.1 months) (Figure 3B). Median PFS2 was 20.8 months (95% CI 15.5-30.6 months) (Figure 3C). Median OS from start of avelumab was 24.3 months (95% CI 20.2-31.8 months) in patients who received any 2L treatment (Figure 3D), and 31.8 months (95% CI 20.6 months-NE) in the 2L EV subgroup, 23.5 months (95% CI 16.9 months-NE) in the 2L PBC subgroup, and 24.3 months (95% CI 15.3 months-NE) in the 2L pembrolizumab subgroup (Figure 3E). Median OS from the start of 2L treatment was 15.1 months (95% CI 13.3-20.1 months) in patients who received any 2L treatment, and 17.8 months (95% CI 11.9 months-NE) in the 2L EV subgroup, 15.1 months (95% CI 11.6 months-NE) in the 2L PBC subgroup, and 15.6 months (95% CI 8.6-21.3 months) in the 2L pembrolizumab subgroup (Supplementary Figure S1A and B, respectively, available at https://doi.org/10.1016/j.esmorw.2025.100646).

In an exploratory analysis in this population without disease progression following PBC, median OS from start of 1L PBC was 38.9 months (95% CI 35.6 months-NE) in the overall population and 31.3 months (95% CI 26.3-38.7 months) in patients receiving any 2L treatment (Supplementary Figure S2A and B, respectively, available at https://doi.org/10.1016/j.esmorw.2025.100646). In subgroups who received 2L EV, PBC, and pembrolizumab, median OS from the start of 1L PBC was 37.2 months (95% CI 26.6-60.6 months), 26.9 months (95% CI 22.2 months-NE), and 25.2 months (95% CI 22.5 months-NE), respectively (Supplementary Figure S2C, available at https://doi.org/10.1016/j.esmorw.2025.100646). Analyses of OS measured from start of 1L PBC should be interpreted with caution because this population only included patients without disease progression following 1L PBC.

In patients who were cisplatin eligible, cisplatin ineligible/platinum eligible, or platinum ineligible, median OS from start of avelumab was 31.2 months (95% CI 20.0 months-NE), 31.8 months (95% CI 22.6 months-NE), and 30.6 months (95% CI 19.5 months-NE), respectively (Supplementary Figure S3A, available at https://doi.org/10.1016/j.esmorw.2025.100646). OS from start of avelumab in subgroups defined by age and primary tumor location are shown in Supplementary Figures S3B-D, available at https://doi.org/10.1016/j.esmorw.2025.100646. In a multivariate Cox regression analysis in the overall population, characteristics associated with OS (*P* < 0.05) were BMI, ECOG PS, and CRP level at the start of avelumab (Table 3).

**Table 3:** Multivariate Cox regression analysis of variables associated with OS from start of avelumab maintenance

Variable	Events/patients	HR (95% CI)	*P* value
Age, years			0.149
<75	59/151	Ref	
≥75	34/119	0.70 (0.43-1.14)	
Sex			0.244
Male	69/201	Ref	
Female	24/69	1.35 (0.82-2.23)	
BMI, kg/m^2^			0.028
<18.5	9/18	1.87 (0.87-4.01)	
≥18.5 and <25	69/194	Ref	
≥25	15/58	0.56 (0.31-1.00)	
ECOG PS			0.008
0	72/221	Ref	
1	17/43	1.29 (0.72-2.31)	
≥2	4/6	5.57 (1.87-16.57)	
Primary tumor location			0.983
Bladder	49/140	Ref	
Renal pelvis	44/127	1.04 (0.67-1.61)	
Urethra	0/3	<0.001	
Histological subtype			0.926
Pure UC	82/234	Ref	
UC with subtype + non-UC	11/36	0.97 (0.50-1.87)	
CRP, mg/dl			0.015
≤0.3	54/185	Ref	
>0.3	39/85	1.70 (1.11-2.61)	
Eligibility			0.563
Cisplatin eligible	31/86	Ref	
Cisplatin ineligible and platinum eligible	54/157	0.77 (0.47-1.27)	
Platinum ineligible	8/27	0.72 (0.29-1.76)	
BOR to 1L PBC			0.216
CR	4/22	0.39 (0.14-1.13)	
PR	48/135	Ref	
SD	41/113	0.96 (0.62-1.48)	
Timing of avelumab initiation			0.892
2021	42/92	Ref	
≥2022	51/178	1.03 (0.75-1.72)	

1L, first line; BMI, body mass index; BOR, best overall response; CI, confidence interval; CR, complete response; CRP, C-reactive protein; ECOG PS, Eastern Cooperative Oncology Group performance status; HR, hazard ratio; OS, overall survival; PBC, platinum-based chemotherapy; PR, partial response; Ref, reference; SD, stable disease; UC, urothelial carcinoma.

In the overall population, the ORR with avelumab maintenance was 25.7% (95% CI 20.8% to 31.0%) and the DCR was 64.8% (95% CI 59.1% to 70.2%). ORR and DCR with any 2L treatment were 37.0% (95% CI 29.2% to 45.4%) and 66.4% (95% CI 58.2% to 74.0%), respectively.

## Discussion

Results from the JAVEMACS chart review study in Japan provide new insights into patient characteristics, treatment patterns, and outcomes in a large population of patients with la/mUC who received avelumab maintenance therapy in a real-world setting.

Patient characteristics in JAVEMACS differed from those of the avelumab arm in JAVELIN Bladder 100,^7^^,^^8^ including a higher median age (73.0 versus 68.0 years; ≥80 years in 16.3% versus 8.0%),^13^ and a higher proportion of patients with an ECOG PS of 0 (81.1% versus 60.9%) and upper urinary tract primary tumors (48.0% versus 30.3%), consistent with other real-world studies in Japan.^10^^,^^14^ JAVEMACS included patients who would have been ineligible for JAVELIN Bladder 100,^7^ including patients who had received 1L ddMVAC (9.1%) and 1-3 (19.4%) or ≥7 (4.6%) 1L PBC cycles, and patients whose treatment-free interval was <4 weeks (27.1%) and >10 weeks (10.3%), reflecting the variation in treatment practices in real-world clinical settings. Compared with patients in the avelumab arm in JAVELIN Bladder 100, a lower proportion of patients in JAVEMACS had CR as best response to 1L PBC (9.1% versus 25.7%) and a higher proportion had SD (39.4% versus 27.7%).^8^ When comparing characteristics at the start of 1L PBC with the start of avelumab maintenance, inflammatory markers such as CRP and neutrophil-to-lymphocyte ratio tended to decrease, and there were no notable changes in CrCl or ECOG PS, suggesting that 1L PBC did not significantly impact renal function or the patient’s general condition. Overall, patient and 1L PBC characteristics were generally similar between the overall population and patients who received 2L treatment.

At data cut-off, the majority of patients were still receiving avelumab maintenance (19.1%) or had received 2L treatment (57.1%). Thus, a minority of patients (23.7%) had discontinued without receiving subsequent treatment, suggesting avelumab maintenance therapy resulted in low levels of ‘patient attrition.’ In the 200 patients who received 2L treatment, EV was the most common subsequent treatment (2L, 66.5%; 3L, 29.8%; 4L, 53.3%), and use of 2L EV increased over time, consistent with the regulatory approval of EV monotherapy following disease progression after prior anticancer therapy in Japan in September 2021.^15^

Median OS from start of avelumab maintenance in JAVEMACS (31.8 months) was longer than that reported for JAVELIN Bladder 100 (23.8 months)^8^ and other real-world studies of avelumab maintenance (21.3-26.2 months).^16^, ^17^, ^18^, ^19^ Multiple factors may have contributed to the longer OS in JAVEMACS, in particular the relatively high proportion of patients receiving 2L treatment and the favorable outcomes in the subgroup receiving 2L EV, which accounted for the majority of 2L treatment received. In subgroups defined by 2L treatment received, median OS from start of avelumab was longest with 2L EV (31.8 versus 24.3 and 23.5 months with 2L pembrolizumab or PBC), consistent with results from the AVENANCE real-world study of avelumab maintenance in France (36.0 versus 16.7 months with 2L EV versus PBC).^19^ Additionally, the lack of observed impact on ECOG PS or CrCl and the reduction of inflammatory biomarkers during 1L PBC may have impacted outcomes. OS results were similar in subgroups defined by primary tumor location and age, including patients aged ≥75 years and ≥80 years, consistent with outcomes reported in older patients in JAVELIN Bladder 100.^13^ Although 28.6% of the JAVEMACS population were cisplatin eligible per published criteria at 1L PBC initiation, 56.0% of patients received 1L cisplatin + gemcitabine. Around a third of patients reduced their platinum dose, suggesting that by modifying treatment, more patients were able to receive cisplatin, irrespective of cisplatin eligibility. OS was consistent in subgroups defined by cisplatin/platinum eligibility, suggesting adequate 1L PBC selection may have resulted in long-term OS. In a multivariate Cox regression analysis, patients with ECOG PS ≥1, BMI <18.5 kg/m^2^, and CRP level >0.3 mg/dl had shorter OS. Similarly other studies of patients with la/mUC in Japan have shown an association between both reduced ECOG PS and low BMI and not receiving further lines of therapy,^20^ and between high CRP and shorter OS,^21^ suggesting that reduced overall health status or inflammation are predictive of a poor prognosis, highlighting the importance of comprehensive patient management. In exploratory analyses of OS from start of 1L PBC in JAVEMACS, median OS in the overall population was 38.9 months, numerically longer than that reported for JAVELIN Bladder 100 (29.7 months)^22^ and other real-world studies, including AVENANCE (26.5 months).^18^^,^^19^

ORR and DCR in JAVEMACS (25.7% and 64.8%, respectively) were numerically higher than those reported in JAVELIN Bladder 100 (14.3% and 50.9%, respectively).^8^ However, results between studies should not be compared directly due to differences in design and populations. The higher ORR observed in this real-world setting may reflect differences in assessment schedules and physician assessments.

The study had several limitations, including the retrospective data collection that relied on existing data in medical records, which may be missing data. Evaluation of disease response may differ at each site. Limitations of analyses of OS measured from start of 1L PBC include the following: analyses were carried out in this selected population who remained progression free after 1L PBC; OS includes time receiving PBC, any interval between PBC and avelumab, and treatment with avelumab and any subsequent therapy; and OS includes immortal time bias because all patients analyzed were required to have survived throughout 1L PBC to be eligible for avelumab treatment. Thus, results should be interpreted with caution. Additionally, analyses of OS from the start of avelumab maintenance in subgroups defined by 2L treatment should be interpreted with caution due to immortal time bias and the limited median observation period from the start of 2L treatment (9.3 months). Although EV was the most common 2L treatment, and outcomes with 2L EV were encouraging, observations were descriptive and potentially confounded, and other real-world biases cannot be excluded. Lastly, the study was conducted in university hospitals and cancer institutes that are likely to have a higher level of expertise in the treatment of la/mUC; therefore, these results may vary from general clinical practice. None the less, this study provides important insights into treatment patterns and outcomes with avelumab maintenance therapy in Japan.

## Conclusions

Overall, results from the JAVEMACS chart review study demonstrate the effectiveness of avelumab maintenance therapy in real-world clinical practice, including in subgroups defined by age, primary tumor location, and cisplatin/platinum eligibility, supporting its recommendation for patients with advanced UC without disease progression after 1L PBC. Additionally, results provide insights into treatment patterns and sequencing in Japan; in this real-world population, a treatment sequence of 1L PBC without disease progression followed by avelumab maintenance and 2L EV appeared to be associated with encouraging long-term outcomes.

## Supplementary Material

Supplementary Material

Supplementary Checklist
