# Supplementary material for: JAVEMACS: a real-world study of avelumab maintenance therapy for advanced urothelial carcinoma in Japan
Source: ESMO Real World Data Digit Oncol. 2025 Nov 20;10:100646. doi: 10.1016/j.esmorw.2025.100646 (PMC13447567; doi:10.1016/j.esmorw.2025.100646)
Supplement: Supplementary file 2 [file mmc1.docx]

**Supplement**

**Supplementary Table S1.** **Baseline characteristics at the start of avelumab maintenance therapy in patients who received 2L therapy**

|  | **Patients who received 2L therapy**  **(n=200)** |
| --- | --- |
| **Age**  Median age (IQR), years  <65 years, n (%)  ≥65 and <75 years, n (%)  ≥75 and <80 years, n (%)  ≥80 years, n (%) | 72 (66-77)  38 (19.0)  91 (45.5)  48 (24.0)  23 (11.5) |
| **Sex, n (%)**  Male Female | 151 (75.5)  49 (24.5) |
| **BMI**  Median BMI (IQR), kg/m^2^  <18.5 kg/m^2^, n (%)  ≥18.5 and <25 kg/m^2^, n (%)  ≥25 kg/m^2^, n (%)  Unknown, n (%) | 22.9 (21.2-25.0)  7 (3.5)  132 (66.0)  46 (23.0)  15 (7.5) |
| **Smoking status, n (%)**  Ever smoker (current or former)  Never smoker  Unknown | 135 (67.5)  57 (28.5)  8 (4.0) |
| **ECOG PS, n (%)**  0  1  ≥2  Unknown | 166 (83.0)  29 (14.5)  2 (1.0)  3 (1.5) |
| **Primary tumor location, n (%)**  Ureter or renal pelvis  Bladder  Urethra | 100 (50.0)  98 (49.0)  2 (1.0) |
| **Metastatic site, n (%)**  Any Regional lymph node Distant lymph node  Visceral  Lung  Liver  Peritoneum  Other organs  Bone  Other | 170 (85.0) 107 (53.5)  63 (31.5)  68 (34.0)  41 (20.5)  18 (9.0)  9 (4.5)  7 (3.5) 39 (19.5) 7 (3.5) |
| **Hemoglobin**  Median (IQR), g/dL  <10 g/dL, n (%)  ≥10 g/dL, n (%)  Unknown, n (%) | 10.4 (9.5-11.5)  74 (37.0)  122 (61.0)  4 (2.0) |
| **NLR**  Median (IQR)  <3, n (%)  ≥3, n (%)  Unknown, n (%) | 2.41 (1.68-3.45)  130 (65.0)  64 (32.0)  6 (3.0) |
| **CRP**  Median (IQR), mg/dL  ≤0.3 mg/dL, n (%)  >0.3 mg/dL, n (%)  Unknown, n (%) | 0.20 (0.08-0.48)  127 (63.5)  66 (33.0)  7 (3.5) |
| **Creatinine clearance**  Median (IQR), mL/min  ≥60 mL/min, n (%)  <60 mL/min, n (%)  Unknown, n (%) | 52.9 (40.7-65.4)  67 (33.5)  123 (61.5)  10 (5.0) |
| **Radical surgery history, n (%)**  Yes  No | 74 (37.0)  126 (63.0) |
| **Timing of avelumab initiation, n (%)**  2021  ≥2022 | 69 (34.5)  131 (65.5) |

2L, second line; BMI, body mass index; CRP, C-reactive protein; ECOG PS, Eastern Cooperative Oncology Group performance status; IQR, interquartile range; NLR, neutrophil-to-lymphocyte ratio.

**Supplementary Table S2. Baseline characteristics at the start of 1L PBC**

|  | **Overall population (N=350)** |
| --- | --- |
| **BMI**  Median (IQR), kg/m^2^  <18.5 kg/m^2^, n (%)  ≥18.5 and <25 kg/m^2^, n (%)  ≥25 kg/m^2^, n (%)  Unknown, n (%) | 23.1 (21.0-25.0)  20 (5.7)  228 (65.1)  81 (23.1)  21 (6.0) |
| **ECOG PS, n (%)**  0  1  ≥2  Unknown | 279 (79.7)  42 (12.0)  7 (2.0)  22 (6.3) |
| **Hemoglobin**  Median (IQR), g/dL  <10 g/dL, n (%)  ≥10 g/dL, n (%)  Unknown, n (%) | 12.0 (10.6-13.3)  45 (12.9)  278 (79.4)  27 (7.7) |
| **NLR**  Median (IQR)  <3, n (%)  ≥3, n (%)  Unknown | 3.13 (2.06-4.69)  150 (42.9)  164 (46.9)  36 (10.3) |
| **CRP**  Median (IQR), mg/dL  ≤0.3 mg/dL, n (%)  >0.3 mg/dL, n (%)  Unknown, n (%) | 0.47 (0.10-2.30)  138 (39.4)  178 (50.9)  34 (9.7) |
| **Creatinine clearance**  Median (IQR), mL/min  ≥60 mL/min, n (%)  <60 mL/min, n (%)  Unknown, n (%) | 52.5 (40.8-68.3)  111 (31.7)  209 (59.7)  30 (8.6) |

1L, first line; BMI, body mass index; CRP, C-reactive protein; ECOG PS, Eastern Cooperative Oncology Group performance status; IQR, interquartile range; NLR, neutrophil-to-lymphocyte ratio; PBC, platinum-based chemotherapy.

**Supplementary Table S3. Characteristics of 1L PBC prior to avelumab maintenance in patients who received 2L therapy**

|  | **Patients who received 2L therapy**  **(n=200)** |
| --- | --- |
| **Cisplatin/platinum eligibility, n (%)**  Cisplatin eligible  Cisplatin ineligible^a^ and platinum eligible  Platinum ineligible^b^  Unknown | 60 (30.0)  106 (53.0)  15 (7.5)  19 (9.5) |
| **1L PBC regimen, n (%)**  Cisplatin + gemcitabine  Carboplatin + gemcitabine  ddMVAC  Other**^c^** | 121 (60.5)  63 (31.5)  14 (7.0)  2 (1.0) |
| **Number of cycles**  Median (IQR)  1-3, n (%)  4, n (%)  5 or 6, n (%)  ≥7, n (%) | 4 (4-4)  41 (20.5)  115 (57.5)  34 (17.0)  10 (5.0) |
| **Platinum dose reduction, n (%)** | 68 (34.0) |
| **First cycle when platinum dose reduction occurred, median (IQR)** | 1 (1-2) |
| **Duration of treatment, median (IQR), weeks**^d^ | 19.1 (14.7-24.1) |
| **Treatment free interval**  Median (IQR), weeks  <4 weeks, n (%)  4-10 weeks, n (%)  >10 weeks, n (%) | 5.1 (3.3-6.7)  59 (29.5)  127 (63.5)  13 (6.5) |
| **BOR, n (%)**  CR  PR  SD | 17 (8.5)  101 (50.5)  82 (41.0) |

1L, first line; 2L, second line; BOR, best overall response; CR, complete response; CrCl, creatinine clearance; ddMVAC, dose-dense methotrexate, vinblastine, doxorubicin, cisplatin; ECOG PS, Eastern Cooperative Oncology Group performance status; IQR, interquartile range; PBC, platinum-based chemotherapy; PR, partial response; SD, stable disease.
^a^Cisplatin ineligibility was defined as a patient who met any of the following criteria: ECOG PS ≥2, CrCl of <60 mL/min, grade ≥2 hearing loss, grade ≥2 peripheral neuropathy, and/or class III or worse New York Heart Association heart failure.^11^

^b^Platinum ineligibility was defined as a patient who met any of the following criteria: ECOG PS ≥3, CrCl of <30 mL/min, or ECOG PS 2 and CrCl of <45 mL/min.^12^
^c^Other 1L PBC regimens included cisplatin + etoposide (n=1) and nedaplatin + gemcitabine (n=1).
^d^1 patient with unknown start and end dates of 1L PBC was excluded from this analysis.

**Supplementary Figure S1. OS from the start of 2L treatment. A.** Overall population. **B.** Subgroups defined by 2L treatment.

**A**

**B**

2L, second line; CI, confidence interval; EV, enfortumab vedotin; NE, not estimable; OS, overall survival; PBC, platinum-based chemotherapy; Pem, pembrolizumab.

**Supplementary Figure S2. OS from the start of 1L PBC*. A.** Overall population. **B.** Patients who received 2L treatment. **C.** Subgroups defined by 2L treatment.

**A**

**B**

**C**

1L, first line; 2L, second line; CI, confidence interval; EV, enfortumab vedotin; NE, not estimable; OS, overall survival; PBC, platinum-based chemotherapy; Pem, pembrolizumab.

*Exploratory analyses of OS measured from the start of 1L PBC in this population of patients without disease progression after 1L PBC should be interpreted with caution. Patients without a start date for 1L PBC were not included.

**Supplementary Figure S3.** **Subgroup analysis of OS from the start of avelumab maintenance. A.** Cisplatin/platinum eligibility. **B.** Age <75 years and ≥75 years. **C.** Age <80 years and ≥80 years. **D.** Primary tumor location.

**A**

**B**

**C**

**D**

1L, first line; CI, confidence interval; Cis, cisplatin; NE, not estimable; NR, not reached; OS, overall survival; Pt, platinum.
